# Potential connection between positive frustration in family leisure time and the promotion of adolescent autonomy

**DOI:** 10.3389/fpsyg.2023.1258748

**Published:** 2023-09-20

**Authors:** Sonia Rivas, Aranzazu Albertos

**Affiliations:** ^1^School of Education and Psychology, Universidad de Navarra, Pamplona, Spain; ^2^Institute for Culture and Society, Universidad de Navarra, Pamplona, Spain

**Keywords:** positive frustration, parental support, family leisure, adolescents, autonomy, coping with stress, structured leisure, motivation

## Abstract

Family relationships during leisure time in adolescence have the potential to promote positive development, particularly in terms of autonomy. However, the scientific literature that links specifically positive family leisure to the development of adolescent autonomy is scarce, and lower when analyzing the role of frustration in leisure time. Grounded in Self-Determination Theory (SDT) this article examines the potential relationship between positive frustration in family leisure time and the promotion of adolescent autonomy. For that purpose, the manuscript addresses four objectives to be discussed consecutively: (1) to delimit the concept of adolescent autonomy and point out the difficulty of parental support; (2) to explore positive frustration, a concept aligned with Csikszentmihalyi’s theory of flow, as a construct that can promote socio-emotional development in adolescence; (3) to describe the components of family leisure; and (4) to understand how the experience of optimal frustration may be linked to the development of adolescent autonomy during family leisure time. From this central question, several additional inquiries emerge: the interplay of frustration and failure in adolescence, the importance of parents and adolescents spending quality time together, the enjoyment in structured family leisure time, the autonomy-supportive parenting in leisure time activities in relation to daily activities, the need to strengthen adolescent bonds developed in infancy, and the complexity of paternal and maternal autonomy granting.

## 1. Introduction

Research focused on the adolescent population emphasizes a model of positive development, which seeks to identify, understand, and strengthen the factors that increase the capacity of adolescents to maintain and improve their health and wellbeing. While health is understood from the combination of social, physical, and mental domains (World Health Organization [WHO], 2006), the concept of wellbeing broadens its hedonic meaning to eudaimonic (Appelqvist-Schmidlechner et al., 2023; Maurer et al., 2023), in that it emphasizes the potential of the person to engage and fulfill oneself, as well as develop and find meaning in one’s life (Ryan and Deci, 2001). Consequently, the approach centered on the vision of a passive adolescent with deficiencies or incapacities who needs protection from risk behaviors (Lerner et al., 2009), has been overtaken by one that considers the adolescent as an active agent and values his or her potential (Seligman and Csikszentmihalyi, 2000; Gagné and Vansteenkiste, 2013).

There is also broad consensus in the literature in defining adolescence as a relational stage in which context plays a crucial role to develop positively (Caldwell and Faulk, 2013; Shek et al., 2019; Belošević and Ferić, 2022a). Bronfenbrenner’s (1979) ecological perspective describes the reciprocal and bidirectional interaction between the different environments in which the individual moves. From a contextual evolutionary paradigm, Lerner’s (1992) work on the contextualization of development reinforces the understanding of developmental regulations, shaped by the connection of ecologies within the community and by the reciprocal influences between adolescents and the changing social context. Also noteworthy is Benson’s (2006) developmental assets approach, which advocates the alignment between what the subject needs and what is offered from the context, thus distinguishing internal from external assets. Finally, Self-Determination Theory (SDT; Ryan and Deci, 2017, 2020) defines different environments between which a mutually beneficial or positive interaction for development can occur. Other authors have taken a similar approach, presenting different spheres for positive personal development (Arranz-Freijo and Barreto-Zarza, 2022). Of all the environments, the ecology of family experience stands out for its importance.

According to SDT assumed in this paper, the family occupies the most privileged position as an educational and socializing context in the lives of adolescents (Kuczynski and Grusec, 1997), because it has the capacity to promote their progress (Ryan and Deci, 2006) –also in leisure time (Coyl-Shepherd and Hanlon, 2013)– through the family relationships. Parents, as a loving and demanding unit, are called upon to ensure comprehensive development, giving priority in adolescence to the development of autonomy, relationships, and competence (Ryan and Deci, 2000; Grusec and Davidov, 2010).

More specifically, family leisure time has the potential to be the space where parents and adolescents respond together to stressors. From the choice of the activity itself, to the behavior during leisure time, or to the management of emotions, work must be done to make these frustrations positive and in line with the development of adolescent autonomy. For this reason, parents bring two of their external developmental assets into play in leisure time: the establishment of clear behavioral rules –which includes setting family boundaries– and the constructive use of time –which includes time at home– (Shek et al., 2019).

The literature has pointed out that leisure experiences in family dynamics are protective factors against risk behaviors, as well as promoters of health and wellbeing. Although there has been a rapid expansion of studies relating to leisure, it is still necessary to further study how positive frustration –specifically in family leisure– helps adolescents develop their autonomy.

For that purpose, and assuming SDT, this manuscript delimits the concept of adolescent autonomy and points out the difficulty of parental support; it shows positive frustration as a variable that promotes socio-emotional development in adolescence; it offers a theoretical body that justifies that positive frustration within family leisure time can contribute to the development of adolescent autonomy; and it finishes with an extensive discussion and conclusions.

## 2. Development of adolescent autonomy and autonomy-supportive parenting

Kimmel and Weiner (1998) defined adolescence as a period of transitions from the end of childhood –marked by physical changes– to the onset of adulthood –identified by the ability to cope with new roles–. The concept of adolescence has been approached from various perspectives. While some paradigms have emphasized biological or cultural determinants, others suggest a combination of biopsychosocial or psychosocial factors (Alonso-Stuyck, 2005). In alignment with this paper, the psychosocial perspective views adolescent autonomy as an integral part of the maturation process, crucial for the development of their initial identity. This process occurs during adolescence, marking the transition into the adult world and playing a vital role in promoting adolescent health and wellbeing (Arnett, 2000).

The trajectory of adolescent development is characterized by a prominent pursuit of greater autonomy. From the perspective of SDT, autonomy is conceptualized as adolescents’ inclination to independently organize their experiences and behaviors. It involves engaging in activities aligned with an integrated sense of self (Deci and Ryan, 2000), all while recognizing the influence and interdependence of established social connections (Ryan and Deci, 2018). Autonomy, regarded as a defining attribute of adolescents’ wellbeing (Avedissian and Alayan, 2021; Bi et al., 2022), encompasses valuational, emotional, cognitive, and behavioral elements that develop at varying rates (Karabanova and Poskrebysheva, 2013). During this phase, individuals aim to form a unique sense of self and identity distinct from parental influences. To construct their identity, adolescents need to establish and organize their abilities, needs, interests, and desires in a manner that allows them to express themselves within a social context. This developmental stage is often described as a “psychosocial moratorium” (Erikson, 1968), where adolescents engage intensely with their environment, treating it as a testing ground for various experiences involving people, objects, or emotions, while temporarily delaying the consequences of their actions.

The process of developing their autonomy inherently intersects with the dynamics of familial interactions and the quality of relationships therein. Consequently, the meaning and significance of adolescent autonomy are profoundly influenced by the quality of these family relationships.

The interrelation between adolescent autonomy and the quality of family relationships constitutes a growing scholarly literature. The examination of the interconnections among adolescents and their parents in the literature is commonly situated within the framework of scrutinizing parental approaches to education and caregiving -parental educational styles vs. parenting practices-. Central to this exploration are the facets of parental warmth and regulatory oversight, both of which hold prominent positions, owing to the foundational role of the family in furnishing adolescents with an environment characterized by affectionate backing, fostering healthy psychological development (Palacios and Rodrigo, 1998). Correspondingly, the dimensions conducive to establishing affirmative emotional bonds during adolescent progression are routinely subjected to examination. Nevertheless, research that comprehensively links educational methodologies to the dimensions underpinning educational enactments within adolescents (Reparaz et al., 2021), while concurrently shaping the trajectory of their conduct with the intent of steering their emotive evolution, remains less abundant in the literature (Steinberg, 2001).

Parental autonomy support is defined as the parent’s active support of the child’s capacity to be self-initiating and independent (Ryan and Deci, 2006). Specifically, the study of parental support for adolescent autonomy has traditionally been carried out from two approaches: separation-individuation vs. SDT (Meeus et al., 2005; Mattanah et al., 2011; Kocayörük et al., 2015). There are two other perspectives that complement the previous view. On the one hand, the promotion of independence seeks to help the adolescent to make decisions for him/herself, prioritizing separation from his/her primary group. On the other hand, the promotion of volitional functioning (Benito-Gomez et al., 2020; Francis and Roemhild, 2022) guides adolescents to behave according to their own interests, values, and beliefs (Soenens et al., 2007), appropriating the decisions of close people and supporting themselves with them. While the former perspective seems to be more prevalent in so-called individualistic cultures, the latter is more aligned with the values of collectivist cultures (Fousiani et al., 2014; Marbell-Pierre et al., 2019).

Autonomy-supportive parenting has been characterized from the perspective of family development as a dynamic process of adaptation, a construct that varies over time, in different life situations (Bumpus et al., 2001; Benito-Gomez et al., 2020) and that adapts to cultural issues (Wang et al., 2017). From this framework, three defining dimensions of autonomy, that interact with each other, are mostly cited (Parra et al., 2015). Firstly, “the adolescent’s ability to act independently and the possibility of knowing how to take control of his or her own life, having first assimilated the meaning itself” (p. 48), refers to *cognitive autonomy*. The educational challenge for the adolescent is to acquire clearer and realistic awareness about himself/herself. Secondly, “the perception of independence through self-confidence and individuality” deals with *behavioral autonomy*. In this process, parents and children must compromise and negotiate, so that the filial decision is exercised in ever-wider areas. Thirdly, “the establishment of more symmetrical affective bonds than those observed during childhood” (Parra et al., 2015, p. 48) relates to *emotional autonomy*. It allows for a better self-awareness and connection with others.

In practice, parent-child relationships that foster adolescent autonomy are complex due to the maladjustments that characterize adolescence, which hinder the relationship and mutual understanding. Adolescents progressively demand greater personal freedom to form and express their opinions (Smetana et al., 2005), seek to assert themselves in their way of thinking and acting autonomously at a time of particular vulnerability, while maintaining emotional ties with their parents (Van Petegem et al., 2013). Also, they seek to satisfy the need to experience risk and progressively share more time with their friends than with their family. Moreover, there is social pressure on families to overprotect their children from possible dangers (Lukianoff and Haidt, 2018), leading to a flight from an upbringing in risk or adversity (Van Petegem et al., 2020). This exercise of hyper parenting has invasive features of hyper protection (Segrin et al., 2013; Millet, 2021; Schønning Vigdal and Kallesten Brønnick, 2022), which may be driven by the adolescent (Brown et al., 2023). Finally, there may also be internal opposition due to the division of opinion between spouses on what conditions should support or disapprove of this autonomy (Caldwell, 2007).

Thus, the development of adolescent autonomy is a fertile ground for disagreements (Steinberg, 2001). Parental disputes are noted in perceptions of conflict in the family (Mastrotheodoros et al., 2020), in the legitimacy of parental authority (Smetana et al., 2005), in the quality of family communication (De Los Reyes et al., 2016) or in the lack of consensus in the choice of shared experiences (Álvarez-Muñoz et al., 2023). Parents navigate between demanding and responsive to the development of adolescent autonomy (Bornstein, 2019). However, parental control and autonomy-supportive behaviors are not shown to be mutually exclusive (Benito-Gomez et al., 2020): low autonomy does not imply greater behavioral control, nor does the absence of control imply autonomy-granting behavior. To sum up, while adolescents seek the development of their autonomy because it is the major aspect of adolescent psychosocial development which occurs within relationships with parents (Steinberg, 1990), parents are called to implement the situational mechanisms involved in the processes of protection and promotion in the development of adolescent autonomy (Kouros and Garber, 2014; Soenens et al., 2018). They are called upon to strengthen their capacities and to protect them from all that can harm them, both from the context and from their own socio-emotional development and growth (Brooks et al., 2016). They must accommodate their external assets of limit control and constructive use of time to their own adolescent’s internal assets, which, in the case of autonomy, are related to five competency areas of their social-emotional development (CASEL, 2017): Self-Awareness, Self-Management, Social Awareness, Relationship Skills, and Responsible Decision-Making.

In essence, the intricate interplay between adolescent autonomy and family relationship quality underscores the significance of recognizing and accommodating the evolving autonomy needs of adolescents within the family structure. In this relationship, parents can help to ensure that experiencing and navigating challenges, setbacks and controlled difficulties fosters the development of autonomy and other essential psychosocial skills during adolescence in ordinary life activities.

## 3. Positive frustration and autonomy development in adolescents

Positive parenting behavior presents warm affective bonds, as well as a structured environment that encourages stimulation and support, freedom from violence. Parents recognize the value of each individual and also empower them to see themselves as active agents who can positively influence others (Rodrigo et al., 2018). Positive parenting (Council of Europe, 2006) in its well-treatment dimension, focuses on the fact that “the family context must offer the conditions that lead to the crystallization of secure attachment bonds with parents while promoting an adaptive contact with adversity” (Arranz-Freijo and Barreto-Zarza, 2022, p. 69).

Adolescents inevitably experience moderate, everyday stress, a phenomenon not unique to this life stage but present throughout the human lifespan. The challenges and stressors adolescents encounter can originate not only from their peer groups but also from the family environment (Van Petegem et al., 2020). Precisely, parents constitute a natural psychosocial stressor, defined as stressors arising from interpersonal relationships or social situations and interactions within the environment (Levi, 1972), and to whom limits should be negotiated. Consequently, the disparity between parental viewpoints and those of adolescents, or differences in their criteria in understanding adolescent autonomy, can lead to conflicts and trigger feelings of frustration (Stroud et al., 2009; Sher-Censor et al., 2011; McKay et al., 2021). However, exposure to adversity does not inevitably result in negative outcomes (Brooks et al., 2019). Thus, living together as a family with adolescents can be a daily source of stress, but it can also serve as a catalyst for positive changes that emerge from coping with stressful situations (Sheridan and Carr, 2020).

Frustration is an emotion that most adolescents experience frequently, and it often prompts them to seek information (Wong, 1979). The concept of frustration, a concept not easy to tackle, is a psychological construct with diverse interpretations depending on the psychological paradigms involved. Traditionally, these interpretations can be distilled into two distinct perspectives: one views frustration as an external impediment to an individual’s pursuit of a specific goal, while the other regards it as a sense of inadequacy or sorrow stemming from the failure to achieve a desired goal (Amsel, 1958; Antunes Ribeiro, 2020). In these situations, adolescent need frustration refers to the actively frustrated needs which, in turn, contribute to ill-being, psychopathology, and maladaptive functioning (Tóth-Király et al., 2019).

Specifically, adolescents experience frustration when the actions and efforts to meet a goal do not produce the expected results (Graesser and D’Mello, 2012). As San Juan and Murai (2022) point out, “frustration occurs when an individual is obstructed from reaching a goal, which leads to escalated efforts to overcome the obstacle, increasing the emotional response with each try.” According to SDT, discomfort arises when the three psychological needs are thwarted, and individuals engage in activities solely to attain rewards or evade punishments. Depending on the intensity of the emotional response, frustration can lead to “aggression, withdrawal, regression, resistance, anger, guilt and remorse, shame, and embarrassment” (Britt and Janus, 1940), making frustration often considered a negative emotion (Maltese et al., 2018).

However, if there is constructive support to help the adolescent work through whatever triggered the frustration, it can become *a source of motivation and curiosity, creating opportunities to move forward*. Each emotion plays a unique role, meaning it provides a response to each situation depending on the factors or elements involved in it. Consequently, emotional factors can influence the development of cognitive resources either positively or negatively. Frustration, as an emotion, when managed appropriately, can provide adolescents with skills to confront situations that challenge our emotional wellbeing (Márquez-Cervantes and Gaeta-González, 2017). Consequently, it can be considered a functional emotion due to its role in aiding adolescents in adaptation. In this scenario, frustration can transform into motivation when the emotional response propels the adolescent to persist in pursuing their goal. Unsatisfied needs create a tension that leads adolescents to an impulse and a search behavior to fulfill the need, thereby reducing the tension and producing satisfaction (García Sanz, 2012). Families, in this context, do not eliminate the arousal; instead, they serve as a stimulus for completing a challenging activity, which increases the adolescent’s need to prove themselves and master the challenge.

Within the theoretical framework of Self-Determination Theory (SDT), frustration is acknowledged as potentially hindering individual growth. However, in a collectivist cultural context (Hofstede, 1984) and drawing on the theory of the *zone of proximal development* (Vygotsky, 1978), parental support and boundaries serve as contextual factors in which adolescents develop autonomy. Parental interactions and communication play a crucial role, providing scaffolding and complementing developmental promotion.

Indeed, understanding the dynamics between frustration and need satisfaction is a flourishing topic. Theoretical models, such as Self-Determination Theory, have been developed that focus on these relationships and have been widely researched.

Some authors (Bartholomew et al., 2011; Warburton et al., 2020; Rodrigues et al., 2021) suggest that need frustration (*dark side* of functioning) and need satisfaction (*bright side* of functioning) are distinct but potentially concurrent constructs (Warburton et al., 2020), identifying different subgroups characterized by different combinations of need satisfaction and need frustration. In fact, it is possible for an adolescent to experience both satisfaction and frustration of the three psychological needs within the same environment, as indicated by Bartholomew et al. (2011). Moreover, absence of need satisfaction does not equal the presence of need frustration (Vansteenkiste and Ryan, 2013). Experiences of frustration regarding a need may not be universally maladaptive if they are also accompanied by feelings of satisfaction related to that need. In any case, experiencing need satisfaction without need frustration represents the most adaptive need profile in the domain of sports (Warburton et al., 2020). Chen et al. (2015) investigated whether satisfaction and frustration of the psychological needs for autonomy, relatedness, and competence, as identified within Basic Psychological Need Theory, contributes to participants’ wellbeing and ill-being. Their results show that the effects of need satisfaction and need frustration were found to be equivalent across four countries and were not moderated by individual differences in the desire for need satisfaction.

Other authors suggest that frustration and need satisfaction are better represented as a single need fulfilment continuum rather than being two distinct and separate constructs (Bidee et al., 2016; Tóth-Király et al., 2019). From this perspective, Tóth-Király et al. (2019) by relying on the framework of Self-Determination Theory, focused on need satisfaction and need frustration of one’s functioning as potential determinants of harmonious passion and obsessive passion across popular screen-based activities. Their findings suggested that general need satisfaction may be a protective factor against the compensatory function of obsessive passion, whereas need frustration may be a potential risk factor.

An adolescent’s response to frustration will depend on their expectations regarding the reward associated with achieving the goal. A balance between effort and reward, whether in the form of prizes or emotions linked to accomplishment, will be necessary to elicit positive stimuli through the activity, particularly in overcoming obstacles and successfully meeting challenges. In the absence of addressing their frustration, families can contribute to a decline in motivation or a loss of confidence in their adolescents, both of which can have adverse consequences.

Therefore, frustration is closely intertwined with an adolescent’s motivational state. The Theory of Basic Psychological Needs is a theoretical framework largely derived from the SDT (Deci and Ryan, 2000). According to this theory, when these three fundamental psychological needs are met (autonomy, competence, and effectiveness), individuals are more likely to experience intrinsic motivation, enjoy greater psychological wellbeing, and exhibit optimal development. In essence, intrinsic motivation, a cornerstone of the SDT, suggests that cognitive and social development serve as a primary source of enjoyment and vitality throughout an individual’s lifespan. However, sustaining and expanding intrinsic motivation requires supportive conditions as it can be easily disrupted by various obstructive contextual factors. Consequently, self-determination facilitated by intrinsic motivation flourishes under conducive circumstances (Ryan and Deci, 2000). According to Ryan and Deci (2000), there is evidence indicating that a foundation of secure and stable relationships is crucial for the expression of intrinsic motivation, with a positive association observed between parent-adolescent relationships and autonomy. On the other hand, frustration of these needs can lead to extrinsic motivation, reduced wellbeing, and adaptation problems. In particular, studies rooted in SDT demonstrated negative effects of need frustration regarding autonomy, competence, and relatedness needs in various contexts such as physical education and voluntary sports (Warburton et al., 2020), classroom education (Cheon et al., 2019), and screen use (Tóth-Király et al., 2019).

On a separate issue, some research suggests that moderate levels of frustration and failure, when approached as learning opportunities, can contribute to the role of frustration and failure in adolescents’ resilience and personal growth (Duckworth et al., 2007; Dweck, 2007; Duckworth and Gross, 2014). This perspective emphasizes the importance of helping adolescents build adaptive coping skills. Resilience is the characteristic that allows one to adapt to stressful events healthily and flexibly (Shek et al., 2019) and to experience positive outcomes from adverse situations (Stainton et al., 2019). Parents should foster those developmental assets—internal and external—that support coping and resilience, rooted in their own characteristics (Staempfli, 2007; Vansteenkiste and Ryan, 2013; Tranter et al., 2021), as well as based on an active education that is characterized by sensitivity, cognitive stimulation, and measured intrusiveness with adversity (Volling et al., 2019). This adaptive coping mechanism aligns with the reconceptualization of the concept of active parental monitoring, defined “as a dyadic process that includes parental solicitation, parental control, and emotional autonomy disclosures” (Brown et al., 2023). This active parental monitoring has been associated with positive adolescent adjustment (Dishion and McMahon, 1998).

Precisely, within the theoretical framework of frustration theories, the concept of frustration tolerance emerges, a concept introduced by Rosenzweig (1944). It pertains to an individual’s inherent inclination to navigate through experiences of frustration while upholding their psychological adjustment intact. This construct encompasses variations in tolerance to frustration across individuals, intricately intertwined with personal attributes and the intensity of encountered stressors. These complex interactions have implications spanning the cognitive and affective domains. Frustration tolerance functions as a metric measuring an individual’s capacity to adapt within their surroundings.

In short, optimal frustration can be considered a necessary complement to successful parenting practice in establishing secure attachments (Arranz-Freijo and Barreto-Zarza, 2022), which accompanies external developmental assets. Specifically, Kohut (1971) delineated the concept of optimal frustration within the psychoanalytic framework as manageable disappointments occurring within the early mother-child relationship. These disappointments facilitate the formation of internal structures, laying the groundwork for self-regulation, and they hold relevance for the subsequent developmental trajectory of the child.

**FIGURE 1:**
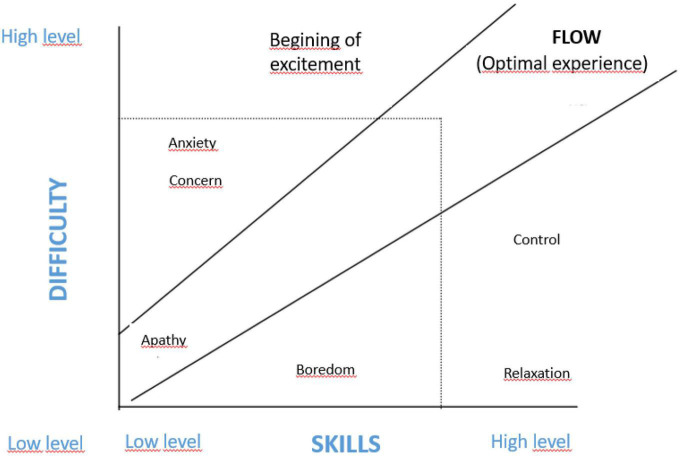
Flow experience adapted from Csikszentmihalyi (Alonso-Stuyck, 2005, p. 102).

Csikszentmihalyi (2008) also introduced this concept and the search for enriching and rewarding experiences from his *flow theory*. A state of flow is experienced when a balance between skill and difficulty is achieved in a challenging activity. Thus, a high degree of concentration, enjoyment, and satisfaction is experienced. Optimal frustration is an essential element in achieving and maintaining this flow state because it provides challenges that stimulate skill development and self-improvement, which, in turn, promotes learning and self-regulation (see Figure 1).

Arranz-Freijo and Barreto-Zarza (2022) have further defined optimal frustration as “the experience, on the part of boys and girls, of adequately timed, dosed, and never traumatic frustrations, which offer attainable challenges that activate the construction of adaptive and successful responses to their environment” (p. 69). The construct of “optimal frustration,” as proposed by Arranz-Freijo and Barreto-Zarza (2022), is situated in the context of childhood and adolescent education within the family environment. In this educational context, parents establish clear boundaries for their children, avoiding overprotection, which is generally associated with negative educational outcomes, and instead, they promote autonomy.

Therefore, and from SDT perspective, optimal frustration can be considered a promoter variable of socioemotional development in adolescence, present in the exercise of fostering resilience and tolerance against adversity. Frustration is not the same as failure, in the sense of a possible outcome of the activity when the goal has not been met.

To sum up, It is suggested that optimal frustration can serve as a potent motivator when recontextualized as a constructive experience that offers valuable learning opportunities. In certain contexts, it may even carry positive implications. Nevertheless, our comprehension of the extent to which frustration contributes to positive experiences remains limited (San Juan and Murai, 2022).

Moving on to other issues, assessing frustration poses a multifaceted challenge. Typically, its evaluation entails the analysis of various variables linked to cognitive-affective responses, encompassing physiological reactions and self-reports. Published studies frequently establish connections between frustration and achievement within the context of learning activities (Cheon et al., 2019: San Juan and Murai, 2022) or in support groups (Sims and Gaugler, 2018; Baucom et al., 2019). These investigations explore how frustration relates to motivation or investment (Bevan et al., 2015), its associations with obstacles (Chou, 2018; Bartlett and Abrams, 2019), and its correlations with difficulties (Cohen, 2022), errors, and discontinuation (Hughes and Morrison, 2018). It is noteworthy that only a limited number of studies explicitly aim to measure frustration.

References in the literature can be found regarding the measurement of challenges rather than frustrations, particularly focusing on effective quality, cognitive activation, and perceptions of achievement (Csikszentmihalyi and Larson, 1987). Additionally, there exists a scale for assessing optimal experiences based on factors such as challenge-skill balance, action-awareness merging, clear goal setting, unambiguous feedback, concentration on the task at hand, a sense of control, loss of self-consciousness, transformation of time, and autotelic experiences (Jackson and Marsh, 1996).

Indeed, there is a substantial body of literature that explores the measurement of brain activation in the context of need frustration (Thetford, 1952; Bierzynska et al., 2016; Ihme et al., 2018), with particular attention to the physiological systems activated in response to perceived stress. Different tools are utilized to experimentally induce stress in adolescents, with the goal of examining their response patterns. These tools include the *Trier Social Stress Test* (TSST) (Allen et al., 2017) and the *Frustration Stress Scale for Adolescents* (FSS-A) (McKay et al., 2021). According to the authors, the FSS-A is an interpersonal psychosocial stress protocol specifically designed for adolescents. Its purpose is to induce moderate frustration in a laboratory setting, achieved through a peer-debate on a value-laden topic identified as potentially frustrating, followed by a serial-subtraction task. The FSS-A protocol spans 90 min, encompassing three phases: Anticipation (involving data collection, personal relevance and frustration rating on value-laden issues, and 5 min for debate preparation); Test (where participants engage in a “judged” 5-min debate followed by an oral subtraction task); and Recovery (involving debriefing and questionnaire completion).

Furthermore, subjective stress levels in adolescents can be assessed using tools such as the *Subjective Stress Scale* (SSS) (Cameron et al., 2017) and the *Perceived Stress Scale* (PSS) (Lakey and Heller, 1988). To measure anger and frustration, researchers often employ the *State-Trait Anger Expression Inventory-2* (Spielberger, 1999). Additionally, *Basic Psychological Need Satisfaction and Frustration Scale* (BPNSFS) *can be used* (Chen et al., 2015; Kuźma et al., 2020). However, it does not fully validate the concept of optimal frustration. The experience of optimal frustration involves a cognitive and emotional complexity that can be challenging to measure accurately.

In the context of autonomy development in adolescents, experiencing positive frustration is thought to be crucial. In any case, the relationship between positive frustration and autonomy development is complex and multifaceted. While some level of challenge is beneficial, excessive frustration without appropriate support can lead to negative outcomes. Therefore, it’s crucial for parents to create an environment where adolescents are provided with opportunities for positive frustration while also receiving guidance, encouragement, and resources to help them navigate challenges effectively.

Family time shared between parents and children, such as leisure time, can be used to develop adolescent autonomy (Joussemet et al., 2008; Lee et al., 2012; Denovan and Macaskill, 2017; Rodríguez-Meirinhos et al., 2020) while achieving this state of flow and creating the environmental conditions that favor the promotion of wellbeing (Iwasaki et al., 2005; Ross et al., 2020; Avedissian and Alayan, 2021). Both parents may manage it differently (Paquette, 2004; Vrolijk et al., 2020), or their competence may be distinctively perceived. Thus, while mothers’ emotional support may be interpreted as a threat to the development of adolescent autonomy, fathers’ emotional support may be understood as autonomy-promoting (Brown et al., 2023). Similarly, perceptions of maternal and paternal promotion of volitional functioning as autonomy granting were significantly negatively associated with both anxiety and depression, whereas promotion of independence as autonomy granting was significantly associated with only anxiety (and not depression) for only perceptions of maternal (and not paternal) parenting (Francis and Roemhild, 2022).

Different meanings are distinguished in the process of coping and adaptation in leisure time: (1) a positive distraction (Waugh et al., 2020), (2) an opportunity to maintain optimism and hope (Liu et al., 2023), (3) a source of normality (Kono, 2015), and (4) a context for positive internal change (Iwasaki et al., 2006). The second and third meanings are mostly studied in vulnerable populations (Hayosh, 2017), while the first and fourth meanings have a more generalized population approach.

Beyond participation, frequency, or type of leisure activities, experiencing positive frustration in family leisure activities should be explored as a mechanism to develop adolescent autonomy.

## 4. Family leisure and adolescent autonomy development

While family leisure research has gained prominence in recent decades, particularly in the European context (Badura et al., 2021), its roots extend back in time. In the 1970s, a pivotal transformation occurred in the field of leisure studies, as researchers began to adopt a psychological perspective to explore personality development. This transformative approach, pioneered by Neulinger (1974), primarily focused on assessing personality within the ideal and valuable context of leisure activities. considering five key dimensions: perceived freedom, intrinsic motivation, extrinsic motivation, end goal, and instrumental goal. Notably, intrinsic motivation was closely associated with classical leisure, where individuals engaged in activities purely for the sake of enjoyment.

Neulinger’s (1981) work emphasized the necessity of promoting leisure as a pathway to human self-realization. He argued for the development of positive attitudes toward leisure, both in and outside of the workplace, with the ultimate goal of creating an environment where the value of leisure equaled that of work. Notably, Neulinger viewed work not as an isolated concept but as interconnected with leisure, laying the groundwork for subsequent research in this area. Building upon Neulinger’s pioneering work, Iso-Ahola (1980) published the first textbook on the subject, highlighting the significance of perceived freedom and intrinsic motivation as fundamental conditions for individuals to truly experience leisure.

As previously stated, Csikszentmihalyi’s (1975, 1996) influential concept of “flow” or optimal experience that occurs when individuals strike a harmonious balance between the challenge of an activity and their skill level, underscored the integration of all facets of life, emphasizing the importance of creatively filling leisure time and finding joy in all endeavors, whether classified as work or leisure. In terms of personal development, Csikszentmihalyi (2001) stressed that individuals must enhance their psychological complexity during both leisure and obligatory tasks to fully benefit from optimal development.

Tinsley and Tinsley (1986) proposed a holistic model for examining leisure, shifting the focus from the activities themselves to the subjective experiences of individuals. This novel psychological approach aimed to capture and categorize how individuals perceive leisure, considering it as an experience influenced by specific conditions and endowed with distinct qualities and benefits. Furthermore, they argued that work-related attitudes could potentially satisfy the seven attributes of leisure, originating from subjective experiences such as concentration, self-forgetfulness, and diminished awareness of time, as well as affective experiences including a sense of freedom, enriched perception of objects and events, heightened emotional intensity, and heightened bodily sensitivity to emotions. Four additional characteristics could also be integrated: freedom of choice, intrinsic satisfaction, provision of optimal stimulation, and a sense of commitment.

Several integrative reviews (Hawks, 1991; Hodge et al., 2015) have synthesized the three themes that family leisure addresses: the promotion of family wellbeing through leisure –where this study is framed–, the costs and constraints of family leisure, and family leisure in marginalized environments.

Leisure is commonly defined as activities undertaken during one’s free time for pleasure and personal choice (Bradley and Inglis, 2012), and it is a source of personal and social growth and development (Alonso et al., 2019). This concept encompasses both an objective perspective, focusing on the activities themselves, the environments in which they occur, and the time periods allocated to them, and a subjective perspective, concerned with the meanings, experiences, and personal needs that these activities fulfill (Codina and Freire, 2020). Measuring leisure involves two distinct approaches: the researcher’s analysis of activity records and the participant’s experiential assessment, which considers aspects like satisfaction and associated meaning (Codina and Freire, 2020).

However, the concept of positive leisure transcends these definitions. It occurs within the context of non-formal education, promoting rest, enjoyment, and the development of social bonds (Da Costa et al., 2020). This form of leisure encourages slow, stable, and sustainable maturation processes that imbue life with meaning (Melton, 2017; Townsend et al., 2017; Kang et al., 2019; Melton and Ellis, 2019). It distinguishes itself from the consumption of experiences characterized by intense, fleeting emotions (Caride, 2012). Adolescents find positive leisure an avenue to fulfill their developmental need for enriching experiences (Caldwell and Weybright, 2018; Rodriguez-Bravo et al., 2020) while simultaneously deterring risky behaviors (Xie et al., 2019).

Moreover, positive family leisure serves as a resource for learning and honing social skills, particularly focusing on strategies for emotional management among adolescents and parents. The concept of family experience ecologies outlines the conditions necessary for achieving this goal (Melton, 2017).

In summary, adolescent family leisure activities can play a pivotal role in personal, social, and emotional development. They can provide opportunities for skill-building, autonomy development, and the maintenance of healthy family bonds, all of which are crucial for adolescents as they navigate the path to adulthood. Nevertheless, it is essential that certain conditions within the family are in place to enable the realization of these benefits during leisure. This involves a thorough consideration of the familial relational and emotional components, alongside communicative aspects.

### 4.1. Relational and emotional component of family leisure

Neither all leisure is equal (Melton et al., 2022) nor is it necessarily a positive experience for the adolescent, because it can be associated with anxiety, stress, loneliness, isolation, and boredom. What sets apart leisure activities with educational benefits for adolescents is the development of emotional competencies and relationships that it facilitates.

There are several leisure activities that adolescents can do with their families, depending on different elements. Firstly, leisure activities may vary the social interaction that they demand, i.e., there are leisure activities in which there is contact with others and those that are autonomous in nature. Secondly, supervision in leisure time can be different. For example, solitary leisure –listening to music or reading–, leisure with the presence of peers (Veenstra et al., 2010), and supervised leisure by adults or peers (Metzger et al., 2009). Thirdly, the amount of effort required in the activity can vary. Thus, active, relaxed, passive, or sedentary leisure can be distinguished (Bradley and Inglis, 2012). Finally, they may differ in their approach. Among the most practised family leisure activities with adolescents are sports, cultural, therapeutic, digital, and naturalistic leisure (Álvarez Muñoz and Hernández Prados, 2023).

However, negotiation and management in leisure time, regardless of the chosen activities, present a similar pattern of parental behavior: the need to negotiate rules, to increase task-focused interactions, and to decrease fragmented interactions in order to achieve higher-quality family leisure (Schwab and Dustin, 2015). Adolescents spend most of their waking time in academic activities or in leisure and free time, both of which are important for the development of qualities such as autonomy or self-control (Caldwell and Smith, 2006; Xie et al., 2017). The predilection for individual leisure activities over family activities increases due to the need for independence that characterizes the adolescent population (Lee et al., 2012). This is probably why the planning and organization of leisure time between parents and adolescents has been overlooked (Marshall et al., 2014).

What is learned within the family context can be extrapolated to others (Melton, 2017). Conflict resolution patterns between adolescents and parents can influence the approaches of the former to resolve conflicts with peers (Van Doorn et al., 2011). Shared leisure and free time between parents and adolescents can be a vital component of family life in which individuals build identity and engage in activities considered “doing family” (Daly, 2001; Shannon, 2022). It has been linked to a higher quality of life (Jenq and Chen, 2021) and higher levels of satisfaction (Agate et al., 2009; Melton and Zabriskie, 2016; Parker et al., 2022), especially among parents (Zabriskie and McCormick, 2003), particularly when fathers are involved (Buswell et al., 2012). However, there is limited literature exploring the development of autonomy within parent-child relationships during leisure time (Larson et al., 2007; Marshall et al., 2014; Van der Eecken et al., 2018; Xie et al., 2019).

Family leisure can be the ideal place to promote positive behavioral and emotional regulation (Da Costa et al., 2020; Álvarez Muñoz and Hernández Prados, 2022), as well as a positive development of adolescents, particularly if there is parental acceptance. When experiencing adverse situations, parents can teach how to cope with them and turn them into opportunities, to favor the learning of values that promote tolerance to frustration and resilience development (Denovan and Macaskill, 2017). If the context, through appropriate frustration, presents engagement, control, and challenge, the adolescents can gain internal control, psychological resilience (Bartko and Eccles, 2003), and thereby improve their sense of autonomy (Maddi, 2013).

Likewise, shared leisure time provides the context in which the family as a primary group can carry out a generational transfer, by sharing behavioral norms with its members and by establishing a value system that enables the basic physical, social, and psychological conditions for adjustment (Bradley and Inglis, 2012; Vukik, 2019). The optimal frustration context creates the condition to protect from negative behaviors or to promote healthy positive development (Caldwell and Faulk, 2013), with parents as role models in their chosen activities (Huebner and Mancini, 2003). Some authors point out that family leisure activities protect from risky behaviors, such as problematic mobile phone use (Albertos and Ibabe, 2021) or alcohol consumption (Albertos et al., 2021), and are associated with engaging in other positive leisure activities with peers (Badura et al., 2017).

In summary, shared family leisure time offers an opportunity to support the holistic development of adolescents (Bradley and Inglis, 2012), fostering communication, socialization, and the transmission of shared values, all while enhancing family cohesion.

### 4.2. Communicative aspects of family leisure in adolescent autonomy development

Parents can influence the development of autonomy in their children during leisure time through their communicative interactions, either by hindering or promoting it. This being so, parent-child relationships based on dialog and negotiation, emphasizing the external or behavioral aspect (Alonso-Stuyck, 2005), are key to adolescent autonomy development. From the establishment of positive emotional autonomy, adequate behavioral autonomy is derived. Consequently, a suitable distance between dependence and independence in parents is needed in relationships allowing the psychosocial maturity of the adolescent.

For the adolescent, shared leisure experiences can help them to develop self-awareness (to become aware of their reactions, bodily sensations, thoughts, beliefs, attitudes, attributions, values, behavior) and to understand how their behavior influences others (Carden et al., 2021). Therefore, cognitive autonomy is specially promoted.

Time spent on family leisure will depend on the climate that is generated, in terms of family cohesion, flexibility, and communication (Ponce de León-Elizondo et al., 2015; Hodge et al., 2018). Communicating different points of view during leisure time opens the door to parental knowledge, through the adolescent’s disclosure (Kerr and Stattin, 2000; Jiménez-Iglesias et al., 2013). Positive family leisure time exhibits the three conditions that define a growth-promoting climate: congruence, empathy, and unconditional positive regard. In leisure time, parent-child relationships must foster adolescent autonomy, characterized by combining affection but also respectful support adolescent’s individuality. The discrepancy between parents’ criteria and children’s opinions about leisure time can create a necessary confrontation but is prescribed for the acquisition of personal judgment as long as the stressful atmosphere is right. The literature has differentiated several ways of coping with stress in adolescents: problem-focused coping and emotion-focused coping. While in the former, stress is reduced by problem-solving, the latter is reduced by distancing. Adolescents approach stress using both approaches (Lee et al., 2012).

To add to this, shared family leisure must create a psychologically safe atmosphere (Duran, 2017; Caldwell and Witt, 2018), in which one’s emotions can be unambiguous, authentic, and henceforth a context that allows one to show one’s own vulnerability. Such vulnerability helps to create bonds with others who are equally vulnerable and who are the first to respect each other’s intrinsic worth (Maurer et al., 2023). Therefore, shared leisure time in which there is assertive communication (Robles et al., 2021) can help parents to be more aware of and attuned to the needs of their adolescent children (Sharp et al., 2006), fostering most notably the promotion of behavioral and emotional autonomy. A favorable communicative environment invites more time together, which can be associated with greater bonding and guaranteed family encounters (Hodge et al., 2017, 2022; Hart et al., 2020; Shannon, 2022), as well as greater protection from adolescent risk behaviors (Albertos et al., 2016) and greater likelihood of adequate parenting skills (Martín Quintana et al., 2018; Van der Eecken et al., 2018).

The challenge at this stage is for the adolescent to become both autonomous and, at the same time, linked to his/her family (Oliva and Parra, 2001). Therefore, manifestations of internal security and self-regulation will be a sign that it is developing positively.

### 4.3. Frustration in family leisure activities and development of adolescent autonomy

It has been mentioned that optimal frustration can represent an opportunity for the emotional development of adolescents during family leisure time. The literature has traditionally categorized leisure activities based on their structure, and it has linked them to the development of autonomy in adolescents (Van der Eecken et al., 2018).

Unstructured activities lack adult supervision and typically take place in public spaces, without a specific skill development objective, but rather emphasizing socialization (Ibabe et al., 2023). Some of the most commonly practised family leisure activities fall into this category, such as gastronomic leisure, digital leisure, and commercial leisure (Álvarez Muñoz and Hernández Prados, 2023). However, these activities receive comparatively less attention in the literature (Marshall et al., 2014).

Research has linked non-structured activities to risk factors in adolescents, including alcohol and substance use (Prieto-Damm et al., 2019), lower academic performance (Badura et al., 2018), deficient inhibitory control goals and skills, and problematic screen use (Ibabe et al., 2023). While some argue that at the very least, non-structured activities do not contribute positively to development (Caldwell, 2008), the effective management of positive frustration in unstructured activities appears to be a more complex challenge for parents.

In contrast, structured leisure activities possess several defining characteristics, including organization, adult supervision, and a focus on skill enhancement (Eccles and Gootman, 2002; Roth and Brooks-Gunn, 2003; Barker et al., 2014). These activities are carefully planned and supervised by adults, providing adolescents with opportunities for learning, skill development, and engaging in meaningful experiences during their free time. This concept aligns with the notion of purposive leisure, emphasizing goal orientation, the promotion of family cohesion, communication, moral values, and the cultivation of healthy lifestyle traits (Shaw and Dawson, 2001).

Family leisure time is the ideal context for the development of autonomy, competence, and relatedness, as long as it is linked to the intrinsic motivation of the adolescent (Larson, 2000; Oropesa, 2014). When these linked actions are present, such as those practiced in family leisure time in a structured and lasting way, they are transformed into joint projects (Young et al., 2001). The concept of “optimal frustration and coping” is particularly relevant in the selection of structured leisure activities, especially during adolescence, which can be a complex stage for negotiating and managing such activities (Marshall et al., 2014). Although parents and children are involved in selecting these activities (Huebner and Mancini, 2003), it is primarily the responsibility of parents to attend to their adolescents’ intrinsic motivation and to make choices in shared activities based on these motivations rather than solely focusing on the structure of the activity itself (Caldwell and Faulk, 2013). Ahmadi et al. (2023) created a classification of 57 teacher behaviors consistent with Self-Determination Theory. They informed that teachers, like parents, could promote autonomy in their adolescent students by providing them with choices instead of giving commands and by providing explanations rather than arbitrary instructions. One’s engagement and involvement can vary as a function of passion for these activities (Tóth-Király et al., 2019). Therefore, parents must be aware of the motivational factors driving adolescents’ participation in leisure activities (Kim et al., 2019; Belošević and Ferić, 2022b) and adjust their expectations by planning activities that align with adolescents’ interests and capabilities (Metzger et al., 2009), such as kinetic activities (Lee et al., 2012), passive leisure (Lee et al., 2017), or home-based digital leisure (López-Sintas et al., 2017). In fact, adolescents with encouraging parents tend to participate more in both organized and unorganized leisure activities (Van der Eecken et al., 2018).

Additionally, the family atmosphere must be conducive to the activities that connect with adolescents’ interests, as adolescents’ attitudes, subjective norms, and perceived behavioral control significantly influence their intention to participate in active family leisure (Taylor et al., 2012; Sanz et al., 2018). When adolescents actively participate in pleasurable leisure activities and are satisfied with them, psychological needs can be fulfilled due to parental monitoring and participation. In fact, tendencies of boredom and Internet addiction behavior can be reduced with encouragement to participate in family and outdoor activities (Lin et al., 2009). Specifically, parental autonomy-granting is generally associated with lower levels of parental encouragement (Van der Eecken et al., 2018). As Valdemoros et al. (2014) mentioned, factors related to internal family functioning are true determinants in the construction of youth physical-sport leisure. In particular, different strategies to promote physical activity among adolescents have been identified, such as increasing levels of family cohesion, parental engagement, parent-child communication, and adolescent self-esteem (Ornelas et al., 2007).

Parents can hinder the development of autonomy during leisure time by creating an environment in which adolescents perceive themselves as overly reliant and dependent on them for support, which is developmentally inappropriate (Steinberg and Silk, 2002). Excessive *amounts of support*, which is one of the two types of parenting practice in leisure time (Xie et al., 2019), does not align with the adolescent’s developmental needs and can hinder his/her perception of parental autonomy support. If parental monitoring in adolescence is generally protective, excessive or harsh monitoring can become intrusive and inhibit psychosocial development (Barber et al., 2005). At its core, what parents are communicating is that the adolescent lacks self-control competence and therefore needs protection (Schønning Vigdal and Kallesten Brønnick, 2022). In this sense, parenting behaviors which are too directive or restrictive with children’s autonomy are associated with lower levels of child physical activity (Jackson et al., 1998; Simons-Morton and Hartos, 2002). Similarly, in an analysis context of parental leisure involvement and its relation to substance use, it is noted that parental leisure overcontrol had a stronger positive relationship with leisure boredom for males than for females (Xie et al., 2019). Ultimately, abundant literature on parenting has positioned itself in a line that suggests that the most beneficial parental behavior for adolescent autonomy development is at a moderate level of control (Karabanova and Poskrebysheva, 2013).

However, psychological control and autonomy granting has to be considered different constructs and not opposite ends of a continuum (Silk et al., 2003), although interrelated (Hauser Kunz and Grych, 2013). Particularly, parental psychological control and autonomy granting exhibited some shared and some unique correlates with indices of child and family functioning. Hierarchical regressions revealed significant interactions between these dimensions, suggesting that the strength of some associations between parents’ use of psychological control and youth adjustment problems depends on the level of autonomy granted exhibited by the parent (Hauser Kunz and Grych, 2013).

*Parental involvement* is known as the second type of parenting practice in leisure time (Xie et al., 2019). Parental monitoring regarding optimal frustration must balance parental responsiveness with parental demandingness, knowing how to distinguish between dependency-oriented and achievement-oriented control (Soenens et al., 2010). Adolescents who perceive their parents as more psychologically and behaviorally controlling report greater psychological distress and less psychological wellbeing (García Mendoza et al., 2019). However, some studies point out these results with this line (Brown et al., 2023). Thus, high levels of some digital interactions between parents and emerging adolescents are potentially perceived as unsupportive of their autonomy. Specifically, greater provision of advice by mothers and greater control by fathers via text messages is interpreted by adolescents as less favorable for their autonomy (Brown et al., 2023). In the same way, adolescents with parents who combine encouragement with autonomy-granting do not necessarily engage more in organized and unorganized leisure activities (Van der Eecken et al., 2018).

Participation in leisure time can provide an opportunity to build positive relationships with others, develop communication and problem-solving skills, and experience a sense of achievement and competence. To do this, parents need to mediate the activity and the context. They must present emotional and motivational support and resources to maintain appropriate involvement, interest, and participation, which involves managing frustration well. Specifically, they can promote the relationship by recognizing and accepting negative emotions instead of punishing them and by showing interest (Ahmadi et al., 2023), they might support competence by providing specific, informative feedback and clear goals. This time must be goal-oriented and/or creative and expressive; it requires discipline and focused attention; it must offer challenges to overcome; it must involve cooperation and interaction with others; it must develop skills and increase competence; and it requires persistence, commitment, and continuity of participation over time (Caldwell, 2008). Knowledge of your adolescent’s coping with stress will help to manage frustration in a positive way. Thus, an active/accommodating coping goal orientation in adolescents will predict participation in structured leisure activities, including shared family time (Hutchinson et al., 2006).

## 5. Discussion

This paper article aims to investigate the relationship between the experience of frustration within the family leisure context and the development of autonomy in adolescents. The central research question focuses on understanding how the experience of optimal frustration may be linked to the development of adolescent autonomy during family leisure time. From this central question, several additional inquiries emerge.

### 5.1. Frustration and failure interplay in adolescence

First and foremost, this document introduces the *intricate interplay between frustration and failure in adolescents.* Frustration and failure frequently intertwine within the experiences of adolescents. When adolescents confront challenges or setbacks in their pursuit of objectives or fulfillment of needs, frustration naturally arises (Warburton et al., 2020; Rodrigues et al., 2021). Moreover, recurrent failures, particularly in domains significant to adolescents, such as academics, sports, or interpersonal relationships, can intensify feelings of frustration. If not effectively managed, this frustration can exert an adverse impact on their psychological wellbeing. Additionally, frustration stemming from failure can significantly influence adolescents’ motivation. Moreover, recent studies have evidenced that parental autonomy granting is linked to adolescents’ life satisfaction. It possesses the capacity to either stimulate perseverance and improvement or induce feelings of hopelessness and diminished motivation. Furthermore, adolescents’ perceptions and responses to frustration and failure are subject to developmental variation. Adolescents are in the process of honing their emotional regulation and coping abilities, which consequently affect how they navigate these experiences.

The focus of this association revolves around the potential negative consequences of an activity when the desired goal remains unattained. It is imperative to underscore that the *essence of this connection does not revolve around conflating frustration with a negation of the need for autonomy or discomfort*—*need satisfaction –.* In other words, and according to different authors (Warburton et al., 2020), the need for frustration and the need for satisfaction are distinct. Instead, it centers on the importance of refraining from equating frustration with autonomy-related concerns, sentiments of coercion and pressure, frustration related to competence and failure, or frustration linked to interpersonal relationships and sentiments of isolation (Rodríguez-Meirinhos et al., 2019, 2020). It is evident, therefore, that the existence of possible optimal frustration is not considered, as suggested in this paper, but rather a frustration in a negative sense and measured as denial of needs, thereby overlooking a potentially positive aspect or, at best, mentioning its positive aspect without delving into further discussion. However, according to the literature, moderate levels of frustration and failure, when perceived as opportunities for learning, can contribute to adolescents’ resilience and personal growth. This perspective underscores the significance of assisting adolescents in cultivating adaptive coping skills.

### 5.2. Adolescent autonomy and optimal frustration from SDT

Related to the previous issue, this paper also evidences the need to *adopt a specific perspective for considering adolescent autonomy in relation to frustration and frame it within a specific stance*. In this case, a notion of autonomy based on the perspective of Self-Determination Theory has been contemplated, wherein social connections and interdependence with others are upheld. The choice is to scrutinize a concept of autonomy where independence from others is sought in an interconnected manner within the system to which the adolescent belongs. Consequently, autonomy is not conceived merely as independence from others or in an isolated manner.

Precisely, as autonomy is an integral part of their maturation process and is pivotal for their identity formation—entailing a desire to achieve independence from others and to orchestrate their own experiences and behaviors (Ryan and Deci, 2000)—it is equally critical to sustain social connections and interdependence with others. Hence, it becomes pertinent to analyze the concept of optimal frustration *from the perspective of the promotion of volitional functioning* (Benito-Gomez et al., 2020; Francis and Roemhild, 2022). As previously mentioned, this standpoint encourages adolescents to act following their own interests, values, and beliefs (Soenens et al., 2007), while also embracing the decisions of close individuals and seeking support from them. Furthermore, it should be noted that according to Soenens et al. (2018), in the context of autonomy as volition, parental promotion of volitional functioning, which includes providing help and advice, is not contradictory to parental norm-setting. Norm-setting involves clear communication of rules, offering meaningful justifications for these rules, and considering the adolescent’s perspective. These authors argue that the introduction of autonomy-supportive norms is effective not only in moral aspects but also in personal domains like friendships. This idea emphasizes that parents can support autonomy while also setting and explaining rules, which can be beneficial for adolescents’ development, including their interpersonal relationships.

Likewise, the consideration of optimal frustration is more congruent *with the values of so-called collectivist cultures* rather than individualistic ones (Fousiani et al., 2014; Marbell-Pierre et al., 2019). Individualistic cultures might place more emphasis on personal autonomy understood as independence from others and minimizing frustration or obstacles in the pursuit of individual goals and happiness. Conversely, in collectivist cultures, there may be a greater emphasis on resilience and personal growth through facing challenges, as this growth can benefit the larger social group or family.

### 5.3. Biopsychosocial approach for measuring frustration

Another issue related to the above is that frustration, as a multifaceted concept, refers to different facets (biopsychosocial), making its global measurement complex. It is necessary to have several valid and reliable instruments to address the measurement of any construct. In this regard, the choice of the instrument will depend on the measurement objectives and the context of the research or evaluation. The theme of frustration is known and studied through various instruments. It is agreed that the experience of frustration generates some type of reaction in adolescents, but this reaction is typically associated with a negative meaning. Thus, it is possible to measure the bodily alterations resulting from experiencing frustration, or the psychological processes that directly impact responses to frustration, such as temperament, emotional responses, resources, and efforts to confront and manage stress (Rosenzweig, 1968). Similarly, it is possible to approach measurements of the social aspect, which refers to family backgrounds, parental responses to the child’s frustration, or stressful events in the social and family environment. Consequently, a biopsychosocial approach is needed to embrace the construct. However, the choice of the instrument also depends on the definition of that construct. The experience of optimal frustration, that encompasses cognitive and emotional intricacies, can be challenging to accurately measure. Nowadays there is no instrument that captures all nuances. Therefore, attempts to approach the measurement of frustration from an achievement perspective remain very scarce.

### 5.4. Importance of parents and adolescents spending quality time together

The text also emphasizes an extensively studied issue in the literature which is the importance of parents and adolescents spending quality time together, highlighting the significance of the context in fostering adolescent positive development (Craig and Mullan, 2012). It underscores how leisure and educational activities during this shared time reflect the values and priorities of society. In a culture heavily influenced by virtual experiences and individualism, where life moves at a rapid pace, it becomes crucial for researchers to consider the potential benefits of family interactions during adolescence. In a world where schedules are often dominated by individual and virtual pursuits, family leisure time offers a valuable opportunity for meaningful interaction, effective communication, and the reinforcement of family ties. Moreover, it can serve as a remedy for feelings of isolation that may arise in an environment characterized by individualistic tendencies.

Support is expressed for the idea that, during adolescence, there is a general increase in autonomy as well as a decrease in relatedness in the relationship with their parents. Furthermore, while the growing sense of autonomy during this period is often associated with a temporary decline of the sense of closeness to parents and increasing conflicts between child and parents, the desire to feel connected to parents is still noticeable (Inguglia et al., 2015).

From a so-called individualistic perspective, it might be tempting to think that parents should foster adolescents’ autonomy simply by allowing them unstructured, free, and rule-free leisure time. However, as previously mentioned, adolescents’ autonomy is not at all incompatible with guidance. Moreover, autonomy from SDT requires mutual influences involving adolescents and their context, in this case, parents, to promote their progress.

### 5.5. Enjoyment in structured family leisure time

Related to the previous question, another idea that underlies the paper and that is consistent with previous studies, is the *educational potential of structured family leisure time during adolescence.* The educational potential of family time during adolescence is important because it considers adolescents’ use of time in a relational context where the family can play an integral role. *Moreover, this leisure time is positively linked with enjoyment.*

It may seem contradictory to think that structured family leisure time can be even *enjoyable* for adolescents. It is a potential that is not incompatible with enjoyment either. However, it is crucial to understand that structure in leisure does not necessarily imply imposition or coercion (Caride, 2012). Furthermore, leisure time should not be a stress-inducing situation, especially concerning how some parents organize activities in leisure time in which there is no choice for improvisation. Structured leisure can offer gratifying experiences in itself and has significant educational potential. In this sense, it is seen as an educational field that has not been fully explored, not only because it can enhance the individual wellbeing of adolescents but also the overall wellbeing of the family.

In summary, structured leisure time with family during adolescence can not only *be enjoyable for adolescents but also has significant educational potential and can contribute to both individual and family wellbeing.* This underscores the importance of valuing and promoting these shared activities in the development of young individuals and the construction of healthy family relationships, enriching the context of leisure activities, facilitating adolescent’s choice of activities, providing supportive control, and mitigating feelings of obligatory participation (Lin et al., 2009).

### 5.6. Granting autonomy in leisure time activities and daily activities

In another vein, the paper initiates a debate on whether parents respond differently to the development of adolescent autonomy during family leisure time compared to other daily activities.

As previously mentioned, support for adolescent parental autonomy is a dynamic and adaptive process that changes over time and varies in different situations but should never be abandoned. According to the literature, there appear to be continuity patterns in the parenting styles used by parents. For instance, parents’ behaviors that undermine adolescents’ autonomy during interactions at the age of 16 were predictive of adolescent hostility as young adults (Allen et al., 2002).

During leisure time, a unique phenomenon occurs, which is the symmetry among players, whether it is a screen-based game or a face-playing game. The parental authority figure, therefore, takes a back seat when it comes to game rules. In this way, everyone is equal during playtime, and the idea of parents as natural stressors can be minimized. Therefore, emotional autonomy can be particularly promoted during playtime for this reason, understood as “the establishment of more symmetrical affective bonds than those observed during childhood” (Parra et al., 2015, p. 48).

However, parents still play a vital role in enhancing their child’s ability to cope with stressors and in understanding that playtime does not involve frustration of basic needs. Shared leisure time is an optimal moment for parents to provide supportive responses to their adolescents, either by focusing on their emotions (consulting with their adolescents or helping them overcome their feelings) or by focusing on the problem (helping them think of ways to solve the problem) (Fabes et al., 2002).

Two characteristics that protect individuals from frustration and aggression can be distinguished: internal, which refers to psychological aspects, and external, which relates to the social component. Family members constitute the external characteristic during these moments, providing security in achievement. This is the difference that especially varies when adolescents are in the presence of other groups, such as their peers, where they must demonstrate their worth to the group to validate their membership. In the case of the internal characteristic, it serves as the framework that provides adolescents with self-awareness in relation to others, as well as tolerance for frustration.

While there is an involuntary/automatic stress response model, parents can help manage what is voluntary/controlled in shared leisure time. It is expected that during adolescence, there may be a tendency to react to stress through negative control strategies, which can lead to maladaptive adjustment by adolescents. Problem-solving is particularly emphasized in family play, where adolescents can promote cognitive autonomy, meaning they decide what to do or not do, regardless of others’ decisions but taking them into consideration. Therefore, in this process, parents can activate primary control coping strategies that aid in emotional development, such as emotional expression, emotional modulation, or emotional-focused social support. Additionally, parents can work on accommodative or secondary control coping, which includes distraction, acceptance, cognitive restructuring, positive thinking, self-encouragement, or minimization during playtime. Lastly, parents should try to avoid passive coping, such as cognitive avoidance, behavioral avoidance, denial, or self-isolation (Compas and Boyer, 2001). Withdrawal or hostility during play can increase tension. If the child is frequently frustrated, quite common in adolescence, they are likely to react with anger or resentment toward others. It is quite likely that some parents may avoid situations in which they know that certain anxiety can be experienced, which is inevitably considered a detrimental feeling. Parents’ positive responses to these emotional situations will become one of the major challenges for families. In any case, sharing this time together, for all the reasons mentioned, leads to positive long-term outcomes. In the face of adolescent frustration, parental resilience and competence are essential to reduce hostile parental reactions toward the adolescent, while parental emotional attributes increase positive parental responses.

### 5.7. Need to strengthen adolescent belonging bonds during the infancy

Connected to the aforementioned point, it is worth noting the need to strengthen adolescent development, balancing structured activities and family bonds, and belonging in infancy. Families recognize the importance of understanding and addressing the specific issues their adolescents face during their leisure time. Their goal is to prevent this free time from becoming a harmful activity for them.

As previously observed, structured leisure activities have been found to yield favorable educational outcomes among adolescents. Nevertheless, adolescents’ time allocation is not exclusively directed toward structured activities; conversely, in a majority of cases, adolescents engage in a combination of both structured and unstructured activities. Over time, with the progression of age, a notable inclination is observed among adolescents to transition away from structured leisure activities. This transition is motivated by a desire to allocate more time to their preferred peer groups, as substantiated by Persson et al. (2007). It is worth noting that unstructured leisure activities often demand lower levels of skill and intrinsic motivation.

It has been established through research, notably by Koutra et al. (2012) and Padhy et al. (2015), that adolescents predominantly focused on unstructured leisure activities tend to exhibit lower levels of subjective wellbeing, heightened substance consumption, and suboptimal academic performance. Nevertheless, it is essential to acknowledge that as adolescents mature, their engagement in unstructured leisure activities becomes an inevitable aspect of their developmental trajectory.

Taking into consideration the constructive impact of structured leisure activities and the demonstrated role of positive familial and domestic sentiments in shielding adolescents from street involvement and delinquent behavior (Persson et al., 2007), it becomes imperative to cultivate—starting in the early years of childhood—attitudes and initiatives that facilitate adolescents’ opportunities to spend quality time with their peers within the familial milieu. Adolescents develop various coping mechanisms to deal with frustration resulting from failure. Some may seek support from family while others might resort to avoidance or other less adaptive coping strategies.

### 5.8. Complexity of paternal and maternal autonomy granting

Determining the best autonomy family granting support during leisure time is presented as a complex task and it is necessary to underline the notion that effective parenting equates solely to fostering encouragement, while ineffective parenting equates solely to granting autonomy, as if these were mutually exclusive concepts.

Understanding the connection between positive frustration and promoting adolescent progress is of paramount importance for parents of adolescents. It involves offering strategies for providing effective support, nurturing resilience, and preventing adverse outcomes, so training parents in need-supporting behaviors to support adolescent autonomous functioning is needed (Meerits et al., 2022). This paper has not addressed the disposition factors associated with adaptive coping when adolescents encounter an optimal frustrating situation, such as the coping strategy of commitment (Connor-Smith et al., 2000; Eschenbeck et al., 2018) or parenting motivational behaviors (Ahmadi et al., 2023). However, It has been mentioned that parental reactions and skills play fundamental roles in successfully facing obstacles, particularly parental empathy and resilience. For instance, research has shown that greater paternal empathy is linked to fathers being more likely to respond positively to their children’s frustrations (Ziv et al., 2020).

Scholars have posited that a balanced negotiation of adolescent autonomy and encouragement within the familial context can engender positive outcomes for both individual adolescents and the family unit as a whole. Leisure time is an ideal moment to showcase those abilities. When the pursuit of autonomy is met with receptive and supportive parental attitudes, a conducive environment for constructive exploration and self-discovery is fostered. This, in turn, can contribute to the cultivation of self-esteem, self-efficacy, and a healthy psychological adjustment among adolescents. Conversely, when the endeavor for autonomy encounters rigidity or overly controlling parental approaches, it may culminate in conflictual family dynamics and impede the establishment of a secure and nurturing familial atmosphere. Such discordant interactions could potentially undermine the development of trust, open communication, and emotional closeness, thereby diminishing the overall quality of family relationships. The key point is parental training. It will be imperative to understand the adolescents’ needs and their world and to adjust accordingly. Additionally, recognizing one’s own parenting competences and helping in training parenting is crucial. Conversely, failure to do so may lead to improperly nurturing the adolescent’s autonomy.

## 6. Limitations

Some limitations should be taken into consideration. Firstly, the paper did not differentiate between the stages of adolescence (early adolescence, middle adolescence, and late adolescence; Gaete, 2015), which exhibit variations in how they approach the central task of identity-seeking. Instead, only a general period of adolescence has been mentioned, neglecting the intrinsic differences of each phase.

Secondly, the adolescent population has been considered uniformly, without delving into the leisure development needs and optimal frustration of young people with disabilities or special needs (Hanna et al., 2005), which also represents a promising line of research. Moreover, adolescents from different cultures have not been considered although culture and context can influence the perception of optimal frustration. Consequently, future research should examine whether differences in distinct adolescent populations affect optimal frustration.

Thirdly, a substantial portion of the reviewed literature covers studies that pose conceptual questions about family leisure and the adolescent population. Indeed, the search for publications containing the word “frustration” in their title or directly considering it as a topic is limited. Numerous articles are retrieved by combining searches involving “failure” and “frustration,” thus associating it with its negative connotations. In contrast, the search using the two main dimensions of parenting, parental responsiveness and parental demandingness (Bornstein, 2019), shed more light on this topic. Thus, given the scarcity of literature regarding frustration and granting autonomy in family leisure time, our analysis was constrained by the scope of the provided information. In some cases, these studies did not capture the complete picture of the experience of optimal frustration concerning family leisure time, especially when the document did not focus on all aspects of the experiences.

Lastly, most of the studies on parenting practices and their influence on adolescent leisure have been focused on families from Western, educated, industrialized, relatively affluent, and developed societies (Xie et al., 2019), being scarce in other contexts. However, this study does not distinguish information on sociodemographic variables in families participating in organized leisure activities and it refers to families in a broad sense. Consequently, information may be overly general.

## 7. Conclusion and recommendations

Research on adolescent development emphasizes the importance of a positive approach, aiming to identify and strengthen factors that contribute to the health and wellbeing of adolescents. Health encompasses social, physical, and mental aspects, while wellbeing also focuses on personal growth and finding meaning in life. This approach considers adolescents as active agents in their development, valuing their potential rather than viewing them as passive individuals with deficiencies. Context plays a significant role in adolescent development, with the family being a crucial socializing and educational environment. Autonomy is an articulated concept of affective, cognitive, and behavioral dimensions. Efforts to set appropriate limits on children’s behavior for the promotion of their autonomy as well as strengthen an optimal and long-lasting relationship in the communicative process are both primary goals for parents in adolescence (Umberson, 1992; Rodríguez-Meirinhos et al., 2020).

In this sense, emerging research on the subject points out that family leisure time can contribute precisely to positive adolescent development, although it is neither the only indicator nor is there a causal relationship between parental relationship and autonomy (Karabanova and Poskrebysheva, 2013).

However, the scientific literature that links specifically positive family leisure to the development of adolescent autonomy is scarce. Added to the aforementioned event is the fact that there is a lack of studies that combine external family assets of limit control and constructive use of leisure time according to Benson’s (2006) developmental assets approach. Consequently, this article outlines how frustration in family leisure time can help the emotional, cognitive, and behavioral promotion of adolescent autonomy.

Lessons learned from this topic show—in the first place—that the role of optimal frustration as a parental support mechanism that helps in the development of adolescents’ autonomy in leisure time remains as an underexplored area. The maturing state of research discusses the resilience to stress through leisure activities (Schneider and Iwasaki, 2003), mentioning coping with stress on a regular basis, usually from an individualistic approach rather than from a family approach (Plancherel et al., 2006; Park and Kim, 2018), and oriented to situations in which the person is particularly vulnerable (Liu et al., 2023). However, there is no mention of optimal frustration as a mechanism that helps parents create and strengthen emotional bonds in regular situations and in settings where interaction with family members occurs on a daily basis and playfully. Therefore, the relationship between shared family leisure and positive frustration research requires further studies that give this issue a central focus and that address it empirically.

Another conclusion that emerges from this study is that, although family relations theory points out that the development of adolescent autonomy is a reciprocal process, it is the parents who play the main role. They are expected to present a climate of affection and trust capable of generating the development of autonomy in the affective, cognitive, and volitional dimensions in adolescence. The role of the affective component of relationships, together with the setting of educational goals aligned with adolescents’ needs, and appropriate educational practices, will help to create a climate that supports positive adolescent development.

In addition to the above, the adolescent developmental trajectory should be analyzed in a contextualized manner. The three fields of adolescent development will overlap and proceed at different paces and at various stages of implementation. In managing family leisure time, parenting should consider the internal dispositions of the adolescent, the resources available to him/her, and the demands experienced and needed for the development of autonomy, in each of its three dimensions. Attention should also be paid to the context in which autonomy develops.

Ultimately, it is essential to understand how parents can best manage the progressive development of their adolescent children’s autonomy in leisure time: their capacity to respond to autonomy, the structure provided, and the level of demand offered. As previously said, parents have the capacity to promote or, otherwise, obstruct their adolescent’s progress. Positive Parenting Programs arising from the Recommendation of the Council of Europe (2006) and managed by specialized professionals (Martínez and Rodríguez, 2023), may be the most effective approach to support families in developing their parenting competences during adolescence, although shared family leisure time is not specifically addressed.

## Author contributions

SR: Conceptualization, Investigation, Supervision, Writing – original draft, Writing – review and editing. AA: Conceptualization, Writing and– review and editing.
